# The Impact of the FIFA 11+ Training Program on Injury Prevention in Football Players: A Systematic Review

**DOI:** 10.3390/ijerph111111986

**Published:** 2014-11-19

**Authors:** Noël C. Barengo, José Francisco Meneses-Echávez, Robinson Ramírez-Vélez, Daniel Dylan Cohen, Gustavo Tovar, Jorge Enrique Correa Bautista

**Affiliations:** 1 Center of Studies in Physical Activity Measurements, School of Medicine and Health Sciences, University of Rosario, Bogotá, DC 111051, Colombia; E-Mails: menesesjose77@gmail.com (J.F.M.-E.); robin640@hotmail.com (R.R.-V.); gustavotovar@hotmail.com (G.T.); jorge.correa@urosario.edu.co (J.E.C.B.); 2 Hjelt Institute, University of Helsinki, Helsinki 00014, Finland; 3 Masira Institute, Faculty of Life Sciences, University of Santander (UDES), Bucaramanga 680006, Colombia; E-Mail: danielcohen1971@gmail.com

**Keywords:** injury prevention, football, FIFA, soccer, neuromuscular performance

## Abstract

The FIFA 11+ is a simple, and easy to implement, sports injury prevention program comprising a warm up of 10 conditioning exercises. The aim of this systematic review was to evaluate the impact of the FIFA 11+ on injury incidence, compliance and cost effectiveness when implemented among football players. MEDLINE, EMBASE and Scopus databases were searched using the search terms “FIFA 11+”, “football”, “soccer”, “injury prevention”, and “The 11”. The titles and abstracts were screened by two independent reviewers and the data were filtered by one reviewer using a standardized extraction form and thereafter checked by another one. The risk of bias and the methodological quality of the studies were evaluated through the PEDro score and Critical Appraisal Skills Programme (CASP). A total of 911 studies were identified, of which 12 met the inclusion criteria of the review. The FIFA 11+ has demonstrated how a simple exercise program completed as part of warm-up can decrease the incidence of injuries in amateur football players. In general, considerable reductions in the number of injured players, ranging between 30% and 70%, have been observed among the teams that implemented the FIFA 11+. In addition, players with high compliance to the FIFA 11+ program had an estimated risk reduction of all injuries by 35% and show significant improvements in components of neuromuscular and motor performance when participating in structured warm-up sessions at least 1.5 times/week. Most studies had high methodological quality and a low risk of bias. Given the large number of people who play football at amateur level and the detrimental impact of sports injuries on a personal and societal level, the FIFA 11+ can be considered as a fundamental tool to minimize the risks of participation in a sport with substantial health benefits.

## 1. Introduction

Football is by far the most popular sport played worldwide. Recent estimates of the International Federation of Football Associations (International Federation of Football Associations; FIFA) suggest that the number of people playing football is close to 270 million [1]. Like most sports, football carries a risk of injury for players, both at professional and amateur level and in all age-groups [2].

The financial loss due to football injuries in the professional English football leagues was estimated to be approximately 118 million euros during the 1999–2000 season [3]. Junge and co-workers calculated the annual costs of football injuries in Switzerland to be approximately 95 million euros in 2003 augmented by the loss of more than 500,000 working days [4]. Finally, the estimated direct and indirect costs of football injuries (medical costs and work absenteeism) in The Netherlands in 2008 were 1.3 billion euros a year [5].

A recent review of the literature revealed that the incidence of injury during football games tended to increase with age across all age groups, with an average incidence of 15 to 20 injuries per 1000 hours of match-play among players older than 15 years [6]. Most injuries (60%–90%) were located in the lower extremities, particularly at the ankle, knee and thigh. Majewski *et al.* [7] conducted a study of sport injuries over a 10-year period and observed 19,530 sports injuries in 17,397 patients. Football accounted for the largest number of injuries; 37% of all injuries reported (7.769 injuries) of which the majority were related to the knee 39.8% [7]. Thus, while there are numerous cardiovascular, metabolic and musculoskeletal benefits associated with participation in football [8,9], it is important to recognize that it is also accompanied by a substantial risk of muscle and ligament injuries.

Multiple modifiable and non-modifiable factors interact to determine injury risk [10,11]. Yet, it is estimated that the majority of time loss in professional football is associated with injuries with modifiable risk factors. There is a substantial literature demonstrating the efficacy of preventive programs which modify these risk factors [12].

One such structured program is the FIFA 11+ injury prevention training program (FIFA 11+), which was developed in cooperation with national and international experts under the leadership of the FIFA Medical and Research Centre (F-MARC), to reduce the incidence of football injuries [13,14]. The FIFA 11+ is a simple and easy to implement sports injury prevention warm-up program comprised of 10 structured exercises which is supported by print and online materials. The FIFA 11+ is the replacement of the FIFA 11 [4,15,16,17,18,19] with a different focus and a number of additions to the program. The program includes exercises which focus on core stabilization, eccentric training of thigh muscles, proprioceptive training, dynamic stabilization and plyometric drills performed with good postural alignment. The program requires no technical equipment other than a ball, and after familiarization can be completed in 10–15 min.

The main aim of this systematic review was to evaluate the impact of the FIFA 11+ on injury risk, and performance in football players. We also evaluated studies examining the economic impact of the program, factors related to the delivery of the program and the influence of compliance.

## 2. Material and Methods

### 2.1. Search Strategy

**Figure 1:**
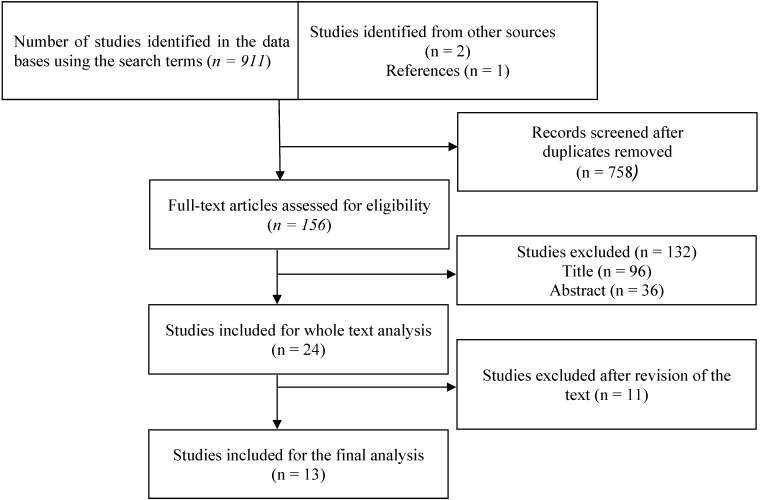
Flow-chart of studies included in the systematic review.

This systematic review incorporates the PRISMA statement (http://www.prisma-statement.org/). Systematic search in electronic databases which were applied by two authors independently. Databases of MEDLINE (via OvidSP) EMBASE (via OvidSP), and Scopus (via ScienceDirect) were searched combining the search terms: “FIFA 11+”, “football”, “soccer”, “injury prevention” and “The 11”. In addition, the reference lists of each publication identified was carefully checked in order to identify additional records. One author (JFME) conducted a search in relevant journals (e.g., *The American Journal of Sports Medicine*, *Journal of Orthopaedic & Sports Physical Therapy*, *Medicine and Science in Sports and Exercise*, *British Journal of Sports Medicine*, *Scandinavian Journal of Medicine* and *Science in Sports*). Furthermore, we only included studies published since 2006 because this is the year in which the program “FIFA 11+” was launched by FIFA with support of the Oslo Sports Trauma and Research Center (OSTRC) and the Santa Monica Orthopaedic and Sports Medicine Research Foundation. No language restrictions were applied. The full search strategies and results for each database can be found in Figure 1.

### 2.2. Inclusion and Exclusion Criteria

Two reviewers independently examined titles and abstracts for relevance; all potentially relevant papers meeting the inclusion criteria were ordered. All full-text papers were then independently screened by two reviewers, with disagreements resolved by consensus, with the participation of a third author where necessary. The inclusion criteria were limited to studies evaluating the effects of the FIFA 11+ and the study designs included were clinical trials, observational cohort and case-control studies. To be included, all studies had to report specific pre and post-intervention outcomes obtained with the acute or chronic implementation of the FIFA 11+. We excluded: (i) studies involving sports other than football; (ii) studies that implemented the 11+ but measured other outcomes than those considered in this systematic review; (iii) case reports; (iv) reviews and editorials. Data extraction involved geographic location; study participants, study design, outcome measures and results, according to the PICO acronym, as displayed in Table 1.

**Table 1:** Inclusion criteria according to the PICO acronym *.

PICO Indicators	Results according PICO
**Design**	Clinical trials and observational studies (cohort and case-control designs)
**Population**	Participants (both male and female) without restriction to a particular age (adolescents, sport players and amateur)
**Intervention**	FIFA 11+
**Comparisons**	Conventional or no warm up
**Outcome measures**	Injury-Incidence rate
	Neuromuscular performance changes
	Cost-effectiveness
	Compliance and method of delivery

* The PICO process (an acronym for patient problem or population (P), intervention (I), comparison (C) and outcome(s) (O)).

### 2.3. Quality Assessment of the Studies

The methodological quality of the randomized controlled trials was assessed using the Physiotherapy Evidence Database scale; a score of 5 of 10 was set as the minimum score for inclusion in the review [20].On the other hand, the methodological assessment of the cohort studies was done by using the Critical Appraisal Skills Programme [21]. The score for each study was determined by two authors. Scores were based on all information available from both the published version and from communication with the authors. Most studies had high methodological quality and a low risk of bias. The randomized controlled trials included had a mean score of 5.6; with the study of Daneshjoo *et al.* [22] the only publication to show a low level of methodological quality (4/10). All observational studies scored between nine and ten out of the eleven points possible. These findings suggest that the current scientific evidence in regard the FIFA’s 11+ program is based predominantly on high quality studies.

## 3. Results

A total of 12 studies implemented in ten different countries were finally included. Approximately half of these studies were conducted in Europe [23,24,25,26,27,28,29], three in North-America [30,31,32,33] and three in Asia [23,33,34]. Eight studies involved male players [19,22,23,24,26,29,32,34], while only five included females [24,26,27,30,31]. The majority of studies were randomized clinical trials [18,22,24,27,28,29,31,33,34], but cohort [23,25,26,30], or an explorative study [22] design was also used.

The outcome measures of the different studies differed substantially. The majority of studies assessed the impact of the FIFA 11+ on injury incidence [25,27,28,30,31] or the acute or chronic effects of the 11+ warm-up on components of neuromuscular performance [22,23,24,26,31,33,34] while others performed an economic evaluation of the FIFA 11+ program, such as the influence of compliance with the program [25,32] or its delivery methods [32].

Table 2 presents the studies which assessed the impact of the FIFA 11+ program on the incidence or number of injuries according to year, country, design, outcome measures and results. Out of the six studies, four reported significantly lower injury incidence [25,27,30,31], while two studies did not find significant reductions in incidence [28,29].

**Table 2:** Summary of the FIFA 11+ studies assessing the impact of the program on incidence or number of injuries according to year, country, design, outcome measures and results.

Source, Year	Participants	Design	Outcomes	Duration, Frequency and Intensity of the Intervention. Period of Implementation	Results
Hammes *et al.* [29], 2014	Male veteran football players (n = 265), mean age 45 years	Randomized controlled trial	Player exposure hours and injuries	Duration: 20 min; Frequency: once/week for 9 months	No significant difference was found between intervention and control group in overall injury incidence (incidence rate ratio (IRR: 0.91 (0.64–1.48); *p* = 0.89). Only severe injuries reached statistical significance with higher incidence in the control group (IRR: 0.46 (0.21–0.97), *p* = 0.04)
Grooms *et al.* [30], 2013	Male collegiate football players (n = 41) 18–25 years-of-age	Cohort study	Lower extremity injury risk and time lost to lower extremity injury	Duration: 20 min; Frequency: 5–6 times/week for 2 seasons	The intervention season had reductions in the relative risk (RR) of lower extremity injury of 72% (RR **^1^** = 0.28; 95% CI **^2^** 0.09–0.85) and time lost to lower extremity injury compared to the previous non-intervention season
Steffen *et al.* [31], 2013	Female youth football players (n = 226) 13–18 years-of-age	Randomized controlled trial	Incidence of all injuries; Neuromuscular performance tests included the Star Excursion Balance Test (SEBT), single-leg balance, triple hop and jumping-over-a-bar	Duration: 20 min; Frequency: 2–3 times/week for 4.5 months	Compared to players with low adherence, players with high adherence to the 11+ had a 57% lower injury risk (RR = 0.43; 95% CI 0.19–1.00). However, after adjusting for covariates, this between group difference was not statistically significant RR = 0.44; 95% CI 0.18–1.06)
Soligard *et al.* [25], 2010	Young female football players (n = 1.055) 13–17 years-of-age	Cohort study	Compliance, injury incidence	Duration: 20 min; Frequency: 1.3 times/week for 10 months	Coaches who had previously utilized injury prevention training coached teams with a 46% lower risk of injury (OR = 0.54; 95% CI 0.33–0.87). Compared with players with intermediate compliance, players with high compliance with the program had a 35% lower risk of all injuries (RR = 0.65; 95% CI 0.46–0.91)
Soligard *et al.* [27], 2008	Young female football players (n = 2.729) 13–17 years-of-age	Randomized controlled trial	Lower extremity injuries (foot, ankle, lower leg, knee, thigh, groin, and hip)	Duration: 20 min; Frequency: 3 times/week for 8 months	In the intervention group there was a significantly lower risk of injuries overall (RR = 0.68; 95% CI 0.48–0.98), lower risk of overuse injuries (RR = 0.47, 95% CI 0.26–0.85) and lower risk of severe injuries (RR = 0.55, 95% CI 0.36–0.83) compared to the control group
Steffen *et al.* [28], 2008	Young female football players (n = 396) 13–17 years-of-age	Randomized controlled trial	Injury incidence and type	Duration: 20 min; Frequency: once/week for 8 months	No effect of the intervention on injury incidence

**^1^** Risk ratio; **^2^** Confidence interval.

The FIFA 11+ leads to significant improvements in thigh muscle strength, jump height, and sprint speed and in a number of measures of balance and proprioception in amateur footballers (Table 3) [22,23,24,26,31,33,34]. Several of these outcomes are relevant to performance within the game, but specific adaptations may also contribute to the reduction of injury incidence observed in other FIFA 11+ studies. Specifically, there is evidence to suggest that the improvements in measures of dynamic balance or proprioception [26,33,34], core-stability, eccentric and concentric hamstrings strength and hamstrings to quadriceps muscle balance [23,26,33,34], are adaptations associated with lower risk for injuries such as anterior cruciate ligament rupture and hamstring [10,11,35] strain.

**Table 3:** Summary of the studies reporting the acute or chronic effects of the FIFA 11+ on performance and physiological measures according to year, country, design, outcome measures and results.

Source, Year	Participants	Design	Outcome	Duration, Frequency and Intensity of the Intervention. Period of Implementation	Results
Bizzini *et al.* [23], 2013	Male amateur football players (n = 20), mean age 25.5 years	Cohort study. ACUTE effects	Acute effects of the FIFA 11+ on performance in 20-m sprints, agility *t*-test, counter- movement jump, squat jump, star excursion balance test and stiffness, quadriceps maximal isometric strength (MVC) and rate of force development (RFD)	Duration: 20 min	Statistically significant changes were found for all the performance variables with the exception of quadriceps MVC and RFD. Significant increases in resting oxygen uptake, core temperature and blood lactate
Impellizzeri *et al.* [24], 2013	Male amateur football players (n = 81), mean age 24 years	Randomized controlled trial	Primary: Dynamic balance: Time-to-stabilization, star excursion balance test, eccentric/concentric flexors strength, core-stability test, vertical jump, 20-m sprint, agility *t*-test	Duration: 20 min; Frequency: 3 times/week for 9 weeks	Statistically significant improvement in time-to-stabilization (−2.8%; 90% CI −4.4–−1.2) and core-stability (−8.9%, 90% CI −14.6–−3.1) in the intervention group compared to the control group. Meaningful but non-significant improvements were observed in eccentric and concentric flexor strength
Nakase *et al.* [33], 2013	Healthy male volunteers (n = 10), mean age: 19 years	Explorative study. ACUTE effects	Acute effect of FIFA 11+ on fluorodeoxy-glucose (FDG) uptake of muscle tissue per unit volume (marker of muscle glucose intake and activation)	Duration: 20 min	Significantly higher FDG accumulation in the rectus abdominus, gluteus medius and gluteus minimus muscles following the FIFA 11+ than in a non-exercising control group
Daneshjoo *et al.* [34], 2012	Young male professional football players (n = 36), mean age 19 years	Randomized controlled trial	Chronic effect of FIFA 11+ on concentric hamstrings:quadriceps ratio (Conventional strength ratio (CSR)), Eccentric hamstrings: concentric quadriceps ratio (Dynamic control ratio (DCR)), and fast:slow speed ratio (FSR) (net peak torque at 300^°^∙s^−1^/net peaktorque at 60^°^∙s^−1^) of the hamstrings and quadriceps	Duration: 20 min; Frequency: 3 times/week for 2 months	Significant increases in DCR in the dominant and non-dominant limb were observed after the 11+ training. In the non-dominant limb, significant improvements were observed in the CSR at 60^°^∙s^−1^ (but not at 180^°^∙s^−1^ or 300^°^∙s^−1^) and in the FSR of the quadriceps (but not in the hamstrings)
Daneshjoo *et al.* [34], 2012	Under 21 year-old football players (n = 36), mean age 19 years	Randomized controlled trial	Joint positioning (proprioception) at 30^°^, 45^°^ and 60^°^ knee flexion. Stork stand test (Static balance) and SEBT (Dynamic balance)	Duration: 20 min; Frequency: 3 times/week for 2 months	Compared to control players who maintained normal activities The program significantly improved joint proprioception at 45^°^ and 60^°^ of knee flexion in the dominant leg and static and dynamic balance
Brito *et al.*, [26], 2010	Sub-elitemale football players (n = 20), mean age 22 years	Cohort study	Isokinetic hamstrings and quadriceps peak torque (PT)	Duration: 20 min; Frequency: 3 times/week for 10 weeks	Hamstrings PT in the non-dominant limb significantly increased by 14.6%, 15.0% and 14.3% during the above contractions/velocities, respectively. In the dominant limb, the concentric PT of the quadriceps increased by 6.9% at 60∙s^−1^, and 8.3% at 180∙s^−1^, whereas that of the hamstrings increased by 20.4% at 60∙s^−1^. The training program significantly increased the H_con_: Q_con_ ratio at 60∙s^−1^ by 14.8% and the H_ecc_: Q_con_ ratio by 13.8% in the non-dominant limb

A significant decrease in dominant leg proprioceptive error from 2.8% to 1.7% at 45^°^ and 60^°^ knee flexion and a significant 10.9% improvement in static balance were observed in the intervention group of one FIFA 11+ study [34]. Impellizzeri *et al*. reported a significant 2.8% improvement in time to stabilization—a measure of dynamic balance. Core stability, which may influence lower extremity biomechanics and in turn anterior cruciate ligament risk during rapid changes of direction [10,35,36], was assessed in two studies, but significant improvements compared to the control groups was only observed in one of these [24].

While the evaluation of strength adaptations in specific muscle groups has been confined to isokinetic testing of hamstrings and quadriceps peak torque or peak torque ratios, increases in fluorodeoxyglucose accumulation in the rectus abdominus, erector spinae, gluteus medius and minimus were reported following the FIFA 11+ indicative of significant activation of these trunk and hip muscles by the program. One of the few studies to have examined the acute effects of the program, Bizzini *et al.* reported statistically significant pre-post warm-up improvements in a number of performance variables, including sprint speed, agility, jump performance and balance.

Soligard et al. [25] reported high compliance with the FIFA 11+ overall and that higher compliance was associated with significantly lower injury risk than intermediate compliance (Table 4). Steffen *et al*., found that delivery of the program by coaches who had been educated during an extensive preseason workshop was associated with greater team adherence to the program compared to its unsupervised delivery [32].

**Table 4:** Summary of studies that assessed the cost effectiveness of the FIFA 11+, compliance with the program or different delivery methods, according to year, country, design, outcome measures and results.

Source, year	Participants	Design	Outcome	Duration, Frequency and Intensity of the Intervention. Period of Implementation	Results
Steffen *et al.* [32], 2013	Female youth football players (n = 226) 13–18 years-of-age	Randomized controlled trial	Comparison of different delivery methods of FIFA 11+ on adherence among female youth football teams	Duration: 20 min; Frequency: 3 times/week for 4 months	Following a workshop delivery of the FIFA 11+ by coaches who had attended a workshop was equally successful with or without the additional field involvement of a physio-therapist. Proper education of coaches during an extensive preseason workshop followed by supervised delivery by these coaches resulted in significantly higher team adherence than an unsupervised delivery of the FIFA 11+
Soligard *et al.* [25], 2010	Young female football players (n = 1.055), 13–17 years-of-age	Cohort study	Interaction between compliance with program and injury incidence	Duration: 20 min; Frequency: 2 times/week for 8 months	Compared with players with intermediate compliance, players with high compliance with the program had a 35% lower risk of all injuries (RR = 0.65; 95% CI 0.46–0.91)

## 4. Discussion

The efficacy of the majority of the FIFA 11+ studies has demonstrated how a simple to implement, exercise-based warm-up program can decrease the incidence of injuries in both male and female amateur football players. In general, teams that implemented the FIFA 11+ had between 30% and 70% fewer injured players [25,27,31]. In addition, high compliance to the FIFA 11+ program was associated with an estimated risk reduction of all injuries by 35% and significant improvements in a number of aspects of motor and neuromuscular performance [25,32].

The efficacy of the FIFA 11+ in decreasing lower extremity injuries concurs with findings in other prevention programs delivered as a warm-up. In a randomized trial among 4.564 Swedish players aged 12 to 17 years, Waldén *et al.* reported that a neuromuscular warm-up program comprised of 6 lower body and trunk conditioning exercises and jump-landing exercises significantly reduced the incidence of anterior cruciate ligament (ACL) injury in female adolescent football players, while a number of other randomized controlled intervention studies also demonstrated that prevention programs targeting football players can reduce the incidence of injuries [37,38,39,40,41].

Of the studies included in the present systematic review, four reported that implementing the FIFA 11+ resulted in a statistically significant reduction in the risk of injury [16,19,28,32] while two studies found no significant effect [28]. The possible explanations for failure to show risk reduction in the study by Steffen *et al.* may relate to the low compliance in the intervention group [28]. Meanwhile, the lack of preventive effects in the study by Hammes *et al.* was likely due to the too low overall frequency of training sessions [29]. Discrepancies in findings could also be explained by differences in population characteristics between the studies. Patterns of injury incidence and injury risk factors are influenced by gender, age and playing level and climatic conditions/geographical location differences [42,43,44,45,46] such that the impact of a standardized program such as the FIFA11+ may vary in different populations. Since, the FIFA11+ is usually implemented as a complete intervention, it is impossible to determine which exercises are the most important in terms of risk reduction when the program is effective.

Nonetheless, while the FIFA 11+ appears to be a useful and effective overall injury risk reduction program at the amateur level, additional loading progressions (volume/intensity) or a greater emphasis on exercises that address specific risk factors may be necessary to produce a significant reduction in injury incidence in higher level players with greater baseline neuromuscular performance. The relative importance of components of the FIFA 11+ are also likely to vary according to player characteristics, in terms of their neuromuscular and biomechanical profile and characteristics such as gender, age and previous injury.

The correct implementation of the FIFA11+ and whether there was direct qualified supervision or not may depend on who conducts the program and the initial training they received. More studies are warranted to further evaluate factors associated with compliance and compare injury prevention and performance of the FIFA 11+ when it is led by qualified coaches compared to other professionals such as strength coaches, sport coaches, team captains, physical therapists, and physiotherapists, [27,30,47] or to unsupervised delivery. While compliance appears to be an important determinant of the effectiveness of the program, there remains limited data describing this relationship [25]. In addition, little is known regarding the influence of compliance and effectiveness of the program in female and senior players. To elucidate potential mechanisms for the programs injury risk reduction effect, future research should also evaluate the impact of the FIFA 11+ on kinematics and kinetics during movements such as cutting or drop jumps, tasks that are used to screen for injury risk, particularly in female athletes, who are at elevated risk of knee ligament injuries [10,36]. In addition, The association between changes in neuromuscular performance variables such as strength, balance and proprioception and reductions in injury risk with a focus on the most frequent and the most severe injuries such as ankle, knee, upper leg and groin injuries.

More than 5000 coaches from approximately forty countries have been taught on how to implement the FIFA 11+ until the present moment [48]. During the following years, FIFA and F-MARC will continue the implementation of the FIFA 11+ with special attention in looking for the best possible cooperation with its member associations of the FIFA 11+ [48].

It is a challenge to convince the coaches of all FIFA member associations to implement the FIFA 11+ and to evaluate its results. However, the experiences of the nationwide implementation of the FIFA 11 in Switzerland are very encouraging, showing how an injury prevention program could be successfully implemented by football coaches with beneficial personal and societal outcomes [4].

## 5. Conclusions

The current evidence suggests that the FIFA 11+ exercise-based warm-up programs can both decrease the incidence of injuries in male and female amateur football players and also improve motor/neuromuscular performance. Evidence of these improvements in performance are important in “marketing” the program to coaches, players and clubs since injury may be seen as a random event [12]. Similarly, for public health agencies to provide economic support for implementation of programs such as the FIFA 11+ evidence of reduction in injury-related costs following implementation of the FIFA 11+ may be important in persuading public health agencies to financially support the delivery of programs such as the FIFA 11+ with the current evidence of cost saving showing promise.

Given the large number of people who play football, the FIFA 11+ and its associated materials could be considered a fundamental public health intervention. The program may have a significant impact in terms of minimizing the potential negative consequences such as direct and indirect healthcare costs and education and productivity time loss [8,9]. Finally, in the delivery of the program, attention should be given to issues of compliance and the proper education of coaches, which appear to be important determinants of the success of the FIFA 11+.
